# Anti-Inflammatory Effect of *Malva sylvestris*, *Sida cordifolia*, and *Pelargonium graveolens* Is Related to Inhibition of Prostanoid Production

**DOI:** 10.3390/molecules22111883

**Published:** 2017-11-03

**Authors:** Cleverson Antonio Ferreira Martins, Michel Leandro Campos, Ana Carolina Irioda, Dile Pontarolo Stremel, Angela Cristina Leal Badaró Trindade, Roberto Pontarolo

**Affiliations:** 1 Department of Pharmacy, Universidade Federal do Paraná, 632 Lothário Meissner Avenue, Curitiba 80210-170, Brazil; cleverson.afm@gmail.com (C.A.F.M.); michelleandocampos@gmail.com (M.L.C.); dile@ufpr.br (D.P.S.); angela@ufpr.br (A.C.L.B.T.); 2 Department of Pharmacy, Pelé Pequeno Príncipe Research Institute, 1632 Silva Jardim Avenue, Curitiba 80250-060, Brazil; anairioda@gmail.com

**Keywords:** anti-inflammatory activity, *Malva sylvestris*, *Sida cordifolia*, *Pelargonium graveolens*, RAW 264.7

## Abstract

The ability of plant extracts and preparations to reduce inflammation has been proven by different means in experimental models. Since inflammation enhances the release of specific mediators, inhibition of their production can be used to investigate the anti-inflammatory effect of plants widely used in folk medicine for this purpose. The study was performed for leaves and flowers of *Malva sylvestris*, and leaves of *Sida cordifolia* and *Pelargonium graveolens*. These are three plant species known in Brazil as Malva. The anti-inflammatory activity of extracts and fractions (hexane, chloroform, ethyl acetate, and residual) was evaluated by quantitation of prostaglandins (PG) PGE_2_, PGD_2_, PGF_2α_, and thromboxane B_2_ (the stable nonenzymatic product of TXA_2_) concentration in the supernatant of lipopolysaccharide (LPS)- induced RAW 264.7 cells. Inhibition of anti-inflammatory mediator release was observed for plants mainly in the crude extract, ethyl acetate fraction, and residual fraction. The results suggest superior activity of *S. cordifolia*, leading to significantly lower values of all mediators after treatment with its residual fraction, even at the lower concentration tested (10 μg/mL). *M. sylvestris* and *P. graveolens* showed similar results, such as the reduction of all mediators after treatment, with leaf crude extracts (50 μg/mL). These results suggest that the three species known as Malva have anti-inflammatory properties, *S. cordifolia* being the most potent.

## 1. Introduction

Inflammatory response is a combination of effects, the occurrence of which is dependent on noxious causes, such as infection and tissue injury [1]. The general effects of the primary cause of inflammation are increased vascular permeability and leukocyte migration to the affected region [2]. Several inflammatory mediators—such as prostaglandins—are released during inflammatory responses. Prostanoids—prostaglandins (PGs) and thromboxane A_2_ (TXA_2_)—are produced from arachidonic acid by enzymes in the cyclooxygenase pathway [3,4]. The inhibition of arachidonic acid metabolism is a well-recognized way to achieve anti-inflammatory action [5] and this mechanism can be observed by measuring levels of the aforementioned products [6].

Plants have been used as medicinal agents for thousands of years and several traditional remedies for the treatment of inflammatory conditions contain plant material [7]. The ability of plant extracts and preparations to reduce inflammation has been proven by different means in experimental models [8] based on their use in folk medicine, showing the importance of studying natural products which are potentially able to be used as disease treatment.

In Brazil, Malva (mallow) is a plant widely used to treat specific conditions. Among dozens of plants popularly known as Malva (mallow), or that contain this word as part of their name, some stand out due to their use in folk medicine, especially *Malva sylvestris* (Malvaceae), known as common mallow, *Sida cordifolia* (Malvaceae) known as white mallow or silky white mallow, and *Pelargonium graveolens* (Geraniaceae) which, despite being from a different family, is known in Brazil as smelling mallow [9] but known as geranium elsewhere. In a recent study [9], it was observed that half of commercial samples purchased as *M. sylvestris* were actually *S. cordifolia*, while 25% of samples purchased as *P. graveolens* were, in fact, *S. cordifolia*. Since these plants are often mistaken or mislabeled, one for the other, and thus used for the same purposes, they were chosen to be compared with each other with regards to their anti-inflammatory properties.

The literature survey shows many identified compounds found in *M. sylvestris*, such as flavonoids (rutin, malvidin 3,5-diglucoside, malvidin 3-*O*-glucoside, malvidin, delphinidin 3-*O*-glucoside, malvidin 3-*O*-(6″-*O*-malonylglucoside)-5-*O*-glucoside, delphinidin, malvidin chloride, genistein, myricetin, apigenin, quercetin and kaempferol), some terpenoids, phenol derivatives (such as 4-hydroxybenzoic acid, 4-methoxybenzoic acid, 4-hydroxy-3-methoxybenzoic acid, 2-hydroxybenzoic acid, 4-hydroxy-2-methoxybenzoic acid, 4-hydroxybenzyl alcohol, 4-hydroxydihydrocinnamic acid, 4-hydroxy-3-methoxydihydrocinnamic acid, 4-hydroxycinnamic acid, ferulic acid and tyrosol), and coumarins (such as 7-hydroxy-6-methoxycoumarin and 5,7-dimethoxycoumarin), in addition to fatty acids and sterols [10,11]. Analysis of *S. cordifolia* have shown the presence of ephedrine and pseudoephedrine, alkaloids (methyltryptophan methyl ester, hypaphorine, 2-(1′-aminobutyl)-indol-3-one, 2′-(3*H*-Indol-3yl-methyl)-butan-1′-ol β-phenethylamine, vasicinol vasicinone and vasicine [12,13]), flavonoids (5,7-Dihydroxy-3-isoprenyl flavone, 5-Hydroxy-3-isoprenyl flavone and the saponins hecogenin) and diosgenin [13,14]. In *P. graveolens*, the compounds found were hydroxybenzoic acid derivatives, hydroxycinnamic acid derivatives, flavonoid aglycones, flavonoid glycosides, flavan-3-ols, dimeric and trimeri-c prodelphinidins [15], myricetin 3-*O*-gluciside-rhamnoside, quercetin 3-*O*-pentoside-gluciside, kaempferol 3,7-di-*O*-gluciside, isorhamnetin aglycone, quercetin 3-*O*-gluciside, rutin, quercetin 3-*O*-pentoside, kaempferol 3-*O*-gluciside, and kaempferol 3-*O*-rhamnoside-gluciside, in addition to aerial parts essential oils (such as β-citronellol, citronellyl formate, and geraniol [16].

*M. sylvestris* use in traditional medicine includes several applications, such as gastrointestinal disturbance, dermatological ailments, menstrual pains, urological disorders, respiratory diseases, and oral diseases. Most of those indications are treated with flower and leaf preparations [10]. In some regions, *M. sylvestris* is among the most important species in terms of medicinal use [17,18]. Recently, it has shown positive results in the treatment of functional constipation in a clinical study conducted with high methodological quality [19]. Animal studies have confirmed its anti-inflammatory activity, but have highlighted the need for further investigation concerning its mechanism of action [20,21]. The main anti-inflammatory results were obtained using aqueous and hydroalcoholic extracts of aerial parts and leaves, respectively [22,23]. The hydroalcoholic extract of leaves was also able to reduce topical inflammation in a mice model [24]. Analysis of the extract showed the main compounds present on it are scopoletin, quercetin and malvidin 3-glucoside, but the authors attributed the observed effect to malvidin 3-glucoside. Leaf extract of *M. sylvestris* showed in vitro anti-inflammatory activity, demonstrated the presence of the compounds scopoletin, caffeic acid and ferulic acid [25].

Similar findings can be attributed to *S. cordifolia*. This plant has been used for a long time in Indian Ayurveda for medicinal properties [26]. Its traditional medicinal indications include inflammatory conditions, such as rheumatism [27]. Studies have shown the anti-inflammatory activity of the aqueous leaf extract and the ethyl acetate fraction of the aerial parts in rat models of edema [12,28]. A hypoglycemic effect was also observed [28]. Ethyl acetate and methanol extracts of aerial parts exhibited significant analgesic activity in mice model [28]. Ethanol extract, chloroform and methanol fractions obtained from *S. cordifolia* showed significant antinociceptive activity on orofacial nociception in mice [29]. Two isolated alkaloids from aerial parts of *S. cordifolia*, 1,2,3,9-tetrahydro-pyrrolo[2,1-*b*]-quinazolin-3-yl-amine and 5′-hydroxymethyl-1′-(1,2,3,9-tetrahydro-pyrrolo[2,1-*b*]-qui-nazolin-1-yl)-haptan-1-one, showed anti-inflammatory and analgesic activities in rat models [30,31]. Conditions involving inflammatory mediators, such as rheumatism, cystitis, lung disorder, fever, and pneumonia, are treated using an infusion, an extract, or a decoction of leaves [13]. Additionally, several different pharmacological activities can be observed for *S. cordifolia*, such as anti-viral, hepatoprotective, anti-ulcer, antidepressive, and neuroprotective, among others [13]. The flavonoids 5,7-dihydroxy-3-isoprenyl flavone and 5-hydroxy-3-isoprenyl flavone were isolated from the chloroform extract of aerial parts of *S. cordifolia*, and both showed anti-inflammatory activity in a rat model comparable to that of phenylbutazone [32].

*P. graveolens* essential oil shows a well-established antimicrobial activity [33] and widespread utilization in the perfumery, cosmetic, and aromatherapy industries [34]. *P. graveolens* medicinal use to treat inflammatory conditions has been claimed in Iran, India, Turkey, and European countries [34]. The hydroalcoholic extract of its flowers has shown wound healing properties in a rat model [35]. In the same animal model, *P. graveolens* methanol extract showed anti-inflammatory effects in colitis treatment [36]. A study conducted with an ethanol extract led to a decrease in inflammatory cell infiltration in a rat model of CCl_4_-induced liver damage, and the authors conclude the hepatoprotective activity was from the flavonoids flavan-3-ols and prodelphinidins [15].

In a literature survey, some studies evaluated the mechanism of the anti-inflammatory action of *M. sylvestris* [11,24,25,37] and *S. cordifolia*, [38], while no studies dedicated to determining the anti-inflammatory mechanism of action of *P. graveolens* or comparing these three species were found. Since they all can be found as Malva, comparing their different extracts and fractions is important since many compounds can be related to the final pharmacological effect in different ways. To investigate the anti-inflammatory mechanism of these plants, the inhibition of prostanoid release from stimulated cells was evaluated, as this will help to better understand the claimed actions of these plants based on their use in folk medicine.

## 2. Results and Discussion

### 2.1. RAW 264.7 Cell Viability

Evaluation of lipopolysaccharide (LPS; 0.5 µg/mL), dexamethasone (10 µM), and a combination of LPS (0.5 µg/mL) and dexamethasone (10 µM) showed no significant effect (data not shown) on cell viability (5 × 10^5^ cells/mL). Extracts of *M. sylvestris*, *S. cordifolia*, and *P. graveolens* were evaluated for their effect on cell viability in the presence of LPS (0.5 µg/mL). For most extracts, the maximum concentration of each extract that did not show significantly (*p* > 0.05) lower cell viability against the control was 50 µg/mL. The only exception was the residual fraction of *S. cordifolia*, for which lower viability was not observed even at the higher concentration tested (1000 µg/mL). Thus, the anti-inflammatory effect of each extract was evaluated at concentrations not higher than 50 µg/mL.

### 2.2. Inflammatory Mediator Release Evaluation

Injury and infection start the inflammatory response, leading to the release of several mediators [1,2]. Among them, products of the arachidonic acid metabolism pathway have been used as biomarkers of anti-inflammatory activity [6], while pathway inhibition is used as a target for anti-inflammatory action [5]. Therefore, the observation of lower values of the release of these products after stimulus indicates anti-inflammatory activity by inhibition of enzymes or antagonism of receptors, related to their production. Arachidonic acid is converted to prostanoids (PGs and TXA_2_) by specific synthases after its conversion to PGH_2_ by PGH synthase—also known as cyclooxygenase—via PGG_2_ [39]. In addition to TXA_2_ the biologically relevant PGs are PGE_2_, PGI_2_, PGD_2_, and PGF_2α_ [3]. Despite their specific functions, all prostanoids are related to inflammatory response, acting as pro-inflammatory, or sometimes as anti-inflammatory, agents depending on the activated receptor, but also in response to inflammatory stimulus. Therefore, a reduction in their levels in the presence of an inflammatory challenge indicates inhibition of the inflammation process [3,40]. Based on that, evaluation of the capacity of different extracts of *M. sylvestris, S. cordifolia*, and *P. graveolens* to inhibit PGs and TXA_2_ production can be considered as confirmation of their potential activity and also as evidence of their possible mechanism of action.

Each extract or positive control (dexamethasone) was compared to a negative control (only LPS 0.5 µg/mL) in order to observe whether they were able to reduce inflammatory mediators such as PGE_2_, PGD_2_, TXA_2_, and PGF_2α_. Since TXA_2_ is highly unstable—being nonenzymatically converted to TXB_2_ (the stable nonenzymatic product of TXA_2_) [41]—the latter is better quantified, and indicates the formation level of the former [42]. The release of inflammatory mediators was evaluated for crude extracts of flowers (only *M. sylvestris*) and leaves, as well as for hexane, chloroform, ethyl acetate and residual fractions obtained from *M. sylvestris*, *S. cordifolia*, and *P. graveolens*. Two concentrations were tested, 10 and 50 µg/mL.

Inflammatory mediator release in LPS-induced RAW 264.7 cells resulted in an increase of 3.63 ± 0.53 for PGE_2_, 6.97 ± 0.84 for PGD_2_, 4.13 ± 0.39 for TXB_2_, and 4.13 ± 0.51 for PGF_2α_. All results are given as the fold-change in relation to basal unstimulated cells (i.e., the level of cell mediator release with no stimulus). On the other hand, dexamethasone showed lower values compared to the LPS-induced group, resulting in 0.12 ± 0.02 for PGE_2_, 0.14 ± 0.002 for PGD_2_, 0.42 ± 0.05 for TXB_2_, and 0.15 ± 0.01 for PGF_2α_, expressed as the fold-change in relation to baseline values. LPS [43,44] and dexamethasone [45] are well established as controls for the RAW 264.7 cell model of inflammation.

In Brazil, *M. sylvestris* is an important medicinal plant, appearing in the fourth edition of the Brazilian Pharmacopoeia [46] and on a recent list of medicinal plant indications published by the Brazilian regulatory agency (National Health Surveillance Agency, ANVISA) [47], where its infusion is indicated by oral route as an expectorant and topically as an anti-inflammatory. Several other uses can be found in the review of Gasparetto and colleagues [10].

**Figure 1:**
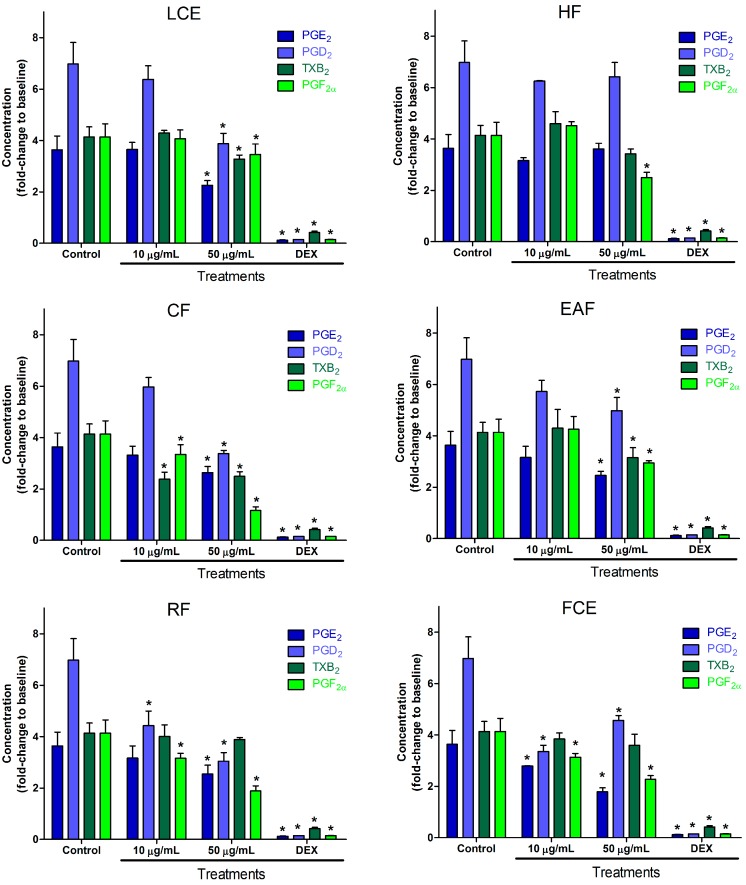
Effect of leaf crude extract (LCE), hexane fraction (HF), chloroform fraction (CF), ethyl acetate fraction (EAF), residual fraction (RF), and flower crude extract (FCE) of M. sylvestris on PGE_2_, PGD_2_, TXB_2_, and PGF_2α_ production in LPS-stimulated RAW 264.7 cells. Cells were treated with LPS in the absence or presence of the fractions at two concentrations (10 and 50 µg/mL). Dexamethasone (DEX) was used as positive control. After incubation, cell culture supernatants were harvested, and PGE_2_, PGD_2_, TXB_2_, and PGF_2α_ production was determined. Values are expressed as mean ± SD. * *p* < 0.001 vs. control (LPS alone).

*M. sylvestris* inhibition of prostanoid production can be seen in Figure 1. The crude extract of leaves showed lower values at 50 µg/mL for PGE_2_, PGD_2_, and TXB_2_. The hexane fraction of leaf extract was not able to inhibit PGE_2_ or PGD_2_, while TXB_2_ and PGF_2α_ release was inhibited, which suggests a concentration of compounds able to inhibit PGF_2α_ in this fraction. Fortunately, all fractions were able to inhibit PGF_2α_ even at lower concentrations (10 µg/mL), as can be observed for the chloroform and residual fractions. Chloroform and ethyl acetate fractions were able to inhibit all tested prostanoids at 50 µg/mL. Complementary to the results observed for leaves, crude extracts of flowers showed statistically (*p* < 0.05) lower values at 50 µg/mL for PGE_2_, PGD_2_, and PGF_2α_, showing that leaves and flowers together are able to inhibit all the prostanoids tested. Similar findings were observed in the literature for *M. sylvestris* in the U937-d cell line [25]. Inhibition higher than 30% was observed for at least one mediator for all extracts or fractions. The highest level of inhibition for each mediator was 50.8% for PGE_2_ by flower crude extracts, 56.4% for PGD_2_ by the residual fraction, and 42.6% for TXB_2_ by the chloroform fraction, which also showed the highest inhibition of PGF_2α_ (71.9%). A previous study has addressed the anti-inflammatory activity of *M. sylvestris* leaves extract with relation to oenin, quercetin, and scopoletin [24], which may be related to observed effects of leaf crude extract (LCE). Unpublished analysis by LC-MS/MS showed that malvin, oenin, and malvidin are present in *M. sylvestris* flowers, while no quantifiable signals were observed in leaves. This may explain the more potent effect of the flower crude extract observed in Figure 1. The activity effect of flowers in the low concentration suggests the presence of specific active compounds, since the richest part in phenols and flavonoids are the leaves [48]. Despite the well-known antagonism in the TXA_2_ receptor [49], flavonoids, such as quercetin and apigenin, are able to reduce TXA_2_ synthesis as well [50]. Figure 1 indicates that similar flavonoids with this property may be concentrated in the chloroform and ethyl acetate fractions, since thromboxane production inhibition was not observed in the aqueous fraction. The particular inhibition of all PG production, without inhibition of TXA_2_ production, in extract of flowers and the aqueous fraction treatments may indicate that the cyclooxygenase (COX)-2 pathway is preferentially blocked [3], perhaps related to the CD44 TXA_2_ stimulation pathway, once this pathway does not enhance PGE_2_ production [51]. Leaf crude extract, and chloroform and ethyl acetate fractions inhibition of all prostanoids indicates the block of a common path for their production. This was observed for apigenin and kaempferol in LPS-induced RAW 264.7 cells by the inhibition of the secretion of proinflammatory cytokine tumor necrosis factor alpha (TNF-α) [52].

**Figure 2:**
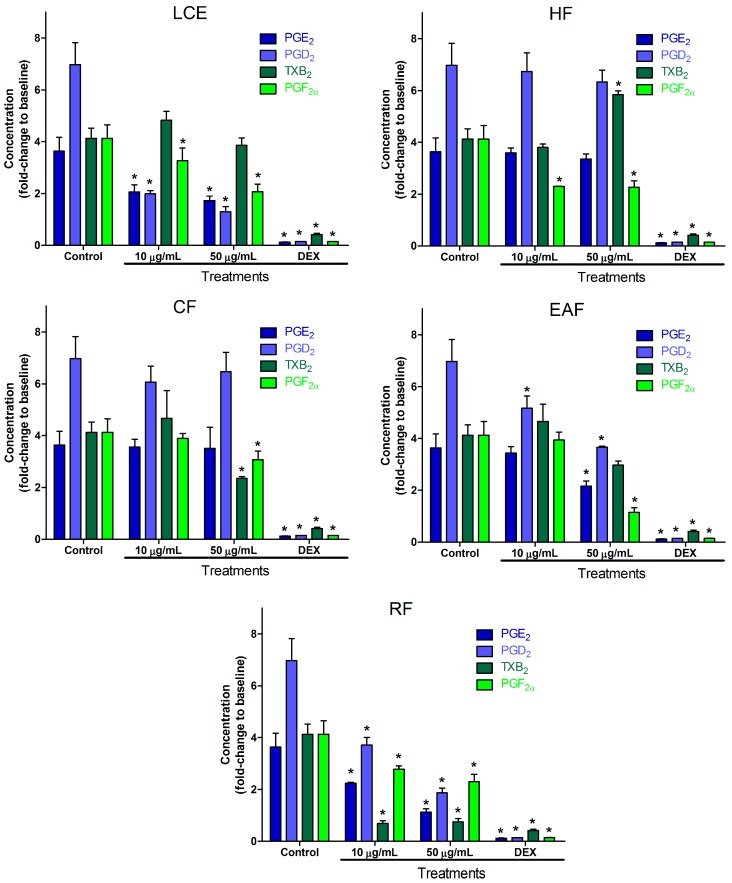
Effect of leaf crude extract, hexane fraction, chloroform fraction, ethyl acetate fraction, and residual fraction of *S. cordifolia* on PGE_2_, PGD_2_, TXB_2_, and PGF_2α_ production in LPS-stimulated RAW 264.7 cells. Cells were treated with LPS in the absence or presence of the fractions at two concentrations (10 and 50 µg/mL). DEX was used as positive control. After incubation, cell culture supernatants were harvested, and PGE_2_, PGD_2_, TXB_2_, and PGF_2α_ production was determined. Values are expressed as mean ± SD. * *p* < 0.001 vs. control (LPS alone).

*S. cordifolia* is widespread in Brazil, but also appears in countries of all continents [13], being used for the treatment of fever [14], rheumatism [53], and throat inflammation [54]. Our results for the anti-inflammatory activity of *S. cordifolia* leaves can be seen in Figure 2. Inhibition of PGE_2_ and PGD_2_ production showed a clear dose–response relationship for the crude extract and residual fraction, suggesting that the compounds responsible for this effect are more polar substances. Still, the residual fraction of *S. cordifolia* led to the reduction of all prostanoids at both concentrations, showing maximum inhibition of 69.08%, 73.18%, 81.87% and 44.25% for PGE_2_, PGD_2_, TXB_2_, and PGF_2α_, respectively. *S. cordifolia* was the only one for which values significantly lower than the control were observed for all prostanoids at 10 µg/mL. The hexane fraction was inhibitory against PGF_2α_ formation at both concentrations, while the chloroform fraction was inhibitory for TXB_2_ and PGF_2α_, but only at the higher concentration level. These indicate that the concentration of polar substances in the crude extract may be useful to improve the efficacy of *S. cordifolia*. This is also well correlated with the most-used preparation, a water infusion [13]. Our in vitro findings confirm the results previously published where aqueous extracts have shown in vivo anti-inflammatory activity related to the PG biosynthesis pathway [12]. The effects on thromboxane production suggests the presence of less polar compounds, such as hexacosanoic acid [26], which are able to stimulate its production in the hexane fraction. Meanwhile, the chloroform fraction may contain concentrated compounds which are able to inhibit thromboxane production. This could have led to an equilibrium of these effects with no statistical difference for TXB_2_ in the crude extract, and finally to high inhibitory effects from the aqueous fraction, where the concentration of hexane soluble compounds is probably very low. The observed antinociceptive action of chloroform and methanolic fractions [29] was related to compounds found in the chloroform extract (5,7-dihydroxy-3-isoprenyl flavone and 5-hydroxy-3-isoprenyl flavones) and ethyl acetate extract (3′-(3″7″dimethyl-2″6″octadiene)-8-*C*-β-d-glucosyl-kaempferol-3-*O*-β-d-glucoside), which have shown anti-inflammatory activity [30,32]. The mechanism of these actions may be related to prostanoid inhibition by the presence of these compounds in the fractions herein tested.

**Figure 3:**
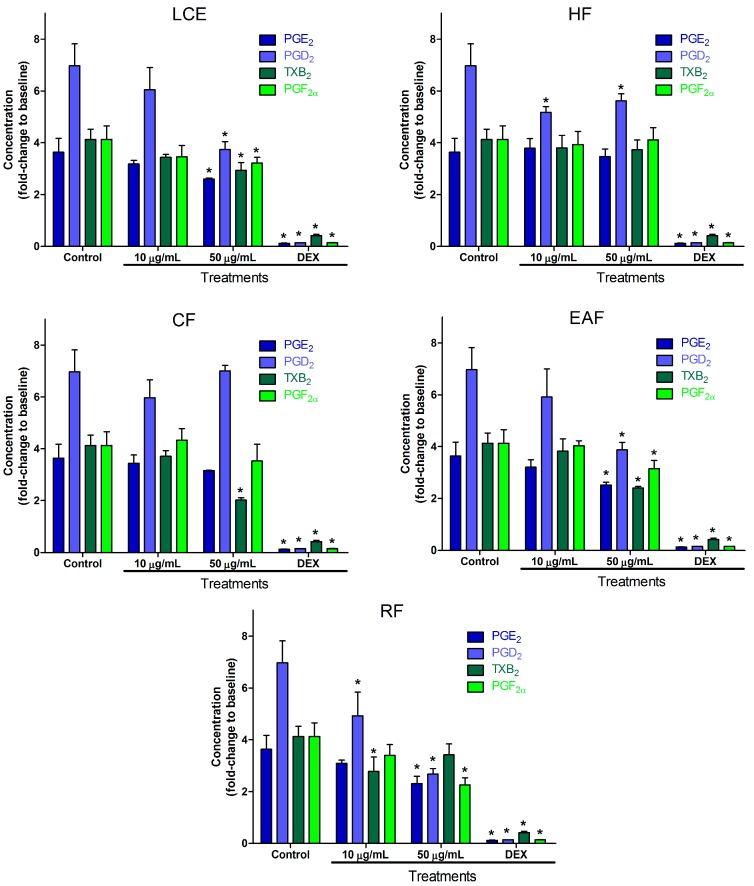
Effect of leaf crude extract, hexane fraction, chloroform fraction, ethyl acetate fraction, and residual fraction of *P. graveolens* on PGE_2_, PGD_2_, TXB_2_, and PGF_2α_ production in LPS-stimulated RAW 264.7 cells. Cells were treated with LPS in the absence or presence of the fractions at two concentrations (10 and 50 µg/mL). DEX was used as positive control. After incubation, cell culture supernatants were harvested, and PGE_2_, PGD_2_, TXB_2_, and PGF_2α_ production was determined. Values are expressed as mean ± SD. * *p* < 0.001 vs. control (LPS alone).

*P. graveolens* is most known for the use of its essential oils [33], even though it has many pharmacological uses [34]. The results of prostanoid inhibition in LPS-induced RAW 264.7 cells by *P. graveolens* can be seen in Figure 3. Some tendency for better efficacy in more polar compounds was observed. Levels of all prostanoids were lower after treatment with 50 µg/mL of crude extract and ethyl acetate fraction, while only TXB_2_ did not show the same significant difference against the control after treatment with 50 µg/mL of residual fraction. The PGE_2_ inhibitory effect of flavonoids present in *P. graveolens*, such as rutin, myricetin, and kaempferol [16], that was observed in LPS-treated rat peritoneal macrophage [55] is in agreement with our findings.

The outcome overview suggests that the anti-inflammatory activity of these plants is related to the cyclooxygenase pathway and is due to polar compounds, mainly in the ethyl acetate and residual fractions. Several findings demonstrate that plant anti-inflammatory activity is related to water-soluble compounds, such as flavonoids [56], anthocyanins [57], tannins [58], and alkaloids [59]. As observed, the three species have some level of pharmacological activity related to anti-inflammatory action. Besides their availability and cultural aspects, their different uses in folk medicine may be related to aspects such as the mode of administration and preparation [60,61,62], or pharmacokinetic properties such as specific tissue distribution [63], or the metabolic stability [64] of some compounds in each extract. Although the use of *P. graveolens* may result in some anti-inflammatory effect, *M. sylvestris* and *S. cordifolia* have shown superior activity in the inhibition of prostanoid production in this model. Still, more studies must be done for *S. cordifolia*, as it showed an even better reduction of prostanoid production than the highly indicated *M. sylvestris*.

## 3. Materials and Methods

### 3.1. Chemical and Reagents

Standards for PGE_2_ (99.0%), PGD_2_ (99.0%), PGF_2α_ (99%), TXB_2_ (99%) and PGB_2_-*d*_4_ (99.0%) were purchased from Cayman (Ann Arbor, MI, USA). Absolute ethanol, n-hexane, acetonitrile, and formic acid were purchased from J.T. Baker (Deventer, The Netherlands). Ethyl acetate and chloroform were purchased from Tedia (Fairfield, OH, USA). Dexamethasone and LPS from *Escherichia coli* were purchased from Sigma-Aldrich (St. Louis, MO, USA). Ultrapure water was obtained using a Milli-Q System (Millipore Corporation, Bedford, MA, USA). High glucose Dulbecco’s modified Eagle’s medium (DMEM) without sodium bicarbonate was purchased from HiMedia (Mumbai, India).

### 3.2. Extracts and Fractions Obtainment

#### 3.2.1. Plant Material and Ethanolic Extract

Standard leaves and flowers of *M. sylvestris* were harvested in a rural region of Ponta Grossa, Paraná, Brazil (25°05′01.24″ S; 50°12′10.84″ W). Leaves of *S. cordifolia* were harvested in a rural region of Dourados, Mato Grosso do Sul, Brazil (22°12′04.12″ S; 54°54′34.26″ W). Leaves of *P. graveolens* were harvested in Curitiba, Paraná, Brazil (25°18′45.87″ S; 49°0′29.4804″ W). The *M. sylvestris* samples were harvested in April, *S. cordifolia* samples were harvested in July, and *P. graveolens* samples were harvested in September. They were authenticated by Prof. Márcia do Rocio Duarte from the Department of Pharmacognosy, Universidade Federal do Paraná, Brazil. The voucher specimens were kept in the herbarium of the Museu Botânico de Curitiba (MBM, Curitiba-Paraná, Brazil). They received the identification numbers MBM voucher #384458, MBM voucher #388190, and MBM voucher #381610 for *M. sylvestris*, *S. cordifolia*, and *P. graveolens*, respectively.

The plant material was dried in a forced circulation oven (Nova Ética, 402) for 24 h at 35 °C, and then crushed in a grinder (IKA, model A-11) in order to obtain adequate granulometry. The extraction was performed by adding absolute ethanol (1:6 *w*/*v*) at room temperature for 7 days, with 30 s agitation twice a day and the sample was protected against light in a closed container. Following that, ethanol extracts were filtered through a 28 µm pore paper filter, then concentrated by low pressure evaporation (Fisatom, 802) and lyophilized (VirTis, Advantage Plus) for 12 h. After lyophilization, the yields (*p*/*p*) were 14.7%, 9.2%, 7.3% and 8.3% for *M. sylvestris* leaves, *M. sylvestris* flowers, *S. cordifolia* leaves, and *P. graveolens* leaves, respectively. These extracts were stored at −40 °C.

#### 3.2.2. Fractions of Leaf Extracts

Crude extracts of leaves (50 g) were resolved in ultra-pure water in a separatory funnel and extracted using 800 mL *n*-hexane. The resulting aqueous phase was further extracted with chloroform (800 mL). Once again, the resulting aqueous phase was extracted with ethyl acetate (800 mL). Therefore, four fractions were obtained: the hexane (HF), chloroform (CF) and ethyl acetate fractions (EAF), and the remaining aqueous phase or residual fraction (RF). The fractions were lyophilized (VirTis, Advantage Plus) and their yields can be seen in Table 1. Crude extracts of flowers were not fractioned due to insufficient available quantity. 

Stock solutions were prepared by dissolving each lyophilized material in absolute ethanol or cell culture medium for a final concentration of 20 mg/mL. Work solutions were prepared by the dilution of stock solutions using cell culture medium in order to reach the appropriate testing concentration.

### 3.3. Analysis of PGE_2_, PGD_2_, PGF_2α_, and TXB_2_

#### 3.3.1. Sample Preparation

Concentrations of PGE_2_, PGD_2_, PGF_2α_, and TXB_2_ in cell culture media were measured after liquid–liquid extraction using ethyl acetate. Internal standard (PGB_2_-*d*_4_, 5 ng/mL), 50 µL of formic acid, and 1 mL of ethyl acetate were added to 1 mL of cell culture media. The mixture was vortex-mixed for 5 min and centrifuged at 20,817× *g* and 10 °C for 5 min. The supernatant (700 µL) was transferred to another polypropylene microtube tube and the ethyl acetate was evaporated to dryness using nitrogen flow. The residue was then reconstituted with a mixture (100 μL) of water, acetonitrile, and formic acid (55:45:0.1 *v*/*v*/*v*). This mixture was centrifuged at 20,817× *g* and 10 °C for 5 min. The new supernatant (70 µL) was transferred to inserts in amber vials and placed inside sample manager drawers. Processed samples were injected into the HPLC system for analysis.

#### 3.3.2. LC-MS/MS

The chromatography system was an Agilent Technologies 1200 series (Palo Alto, CA, USA), which was coupled to an AB Sciex API 3200 triple quadrupole mass spectrometer (Toronto, ON, Canada) equipped with an electrospray ionization (ESI) source. The separation was performed on a Zorbax Eclipse XDB-C18 column (4.6 × 50 mm; 1.8 μm) at 40 °C. The mobile phase solvents were water (A) and acetonitrile (B), both containing 0.1% formic acid at a flow rate of 0.7 mL/min. The initial mobile phase composition was maintained at 55% A for 0.5 min, changed linearly to 25% (0.5–1.57 min), then followed by a return to the initial condition in 1.58 min and kept until 4 min (total running time) for the chromatograph column equilibrium. The injection volume was 5 µL. Mass spectrometry detection was performed in negative ionization mode using multiple reaction monitoring. The transitions used for quantitation were 351 > 271 for PGE_2_ and PGD_2_, 353 > 193 for PGF_2α_, and 369 > 169 for TXB_2_. The internal standard transition was 337 > 113. The curtain, nebulizer, auxiliary, and collision gases were set at 10, 50, 40, and 10 psi, respectively, while the ion spray voltage and source temperature were set at −4.5 kV and 450 °C, respectively. The declustering potential, entrance potential, collision cell entrance potential, collision energy, and collision cell exit potential were optimized at −25, −5, −20, −22, and −20 V respectively for PGE_2_ and PGD_2_; −50, −4.5, −20, −30, and −4 V respectively for PGF_2α_; −35, −5, −20, −24, and −2 V respectively for TXB_2_; and −45, −4.5, −20, −34, and 0 V respectively for the internal standard. Analyst^®^ software (version 1.4.2, AB Sciex, Foster City, CA, USA) was used for data acquisition. The applied method was linear for all analytes between 5 ng/mL (lower limit of quantitation) and 200 ng/mL with a correlation coefficient higher than 0.99. Within-run and between-run precision as relative standard deviation were less than 12.4% and 8.4%, respectively, while within-run and between-run accuracy were 95–109% and 94–106%, respectively [65].

### 3.4. Pharmacological Assessment

#### 3.4.1. Cell Culture

Murine macrophage cell line RAW 264.7, obtained from the Paul Ehrlich Technical and Scientific Association (UFRJ, Rio de Janeiro, Brazil), was cultured in high glucose DMEM without sodium bicarbonate, supplemented with 10% fetal calf serum, L-glutamine, penicillin (300 mg/mL), and streptomycin (50 mg/mL). Cells were grown at 37 °C under 5% CO_2_ in fully humidified air. Before each experiment, cells (5.0 × 10^5^ cells/mL) were plated in 24-well plates.

#### 3.4.2. Effect of Extracts and Fractions on Cell Viability

Cell viability was determined after a 24 h incubation with each extract or fraction at several concentrations in order to find out their toxic concentration in the presence of 0.5 µg/mL LPS. The chosen concentrations for each extract or fraction were 1.0, 10.0, 50.0, 100.0 and 1000.0 µg/mL. The viability was determined through MTT assay. The RAW cells were incubated with MTT solution for 4 h. After incubation, the supernatant was removed, the formazan crystals were dissolved in 500 µL of DMSO, and absorbance at 540 nm was determined. Absorbance of non-treated cells (control) was considered as 100% viability. Each assay was performed in three independent replicates.

#### 3.4.3. Inflammatory Mediator Release Evaluation

Extracts or fractions were evaluated using LPS to stimulate the release of inflammatory mediators from cells (5.0 × 10^5^ cells/mL) after 24 h incubation at 37 °C under 5% CO_2_ in fully humidified air. Crude extract or fraction activity was assessed at 10 and 50 µg/mL. Dexamethasone (10 µM) was used as a positive control due to its well-established anti-inflammatory effect on RAW 264.7 cells stimulated by lipopolysaccharide [66,67] and its previous use in anti-inflammatory evaluation of plant material [68,69]. Baseline levels were obtained in the absence of treatment or LPS induction. Measurement of inflammatory mediators PGE_2_, PGD_2_, TXB_2_, and PGF_2α_ in cell culture medium was performed using the aforementioned LC-MS/MS method.

#### 3.4.4. Statistical Analysis

All experimental values are presented as mean ± SD. Treated groups (extracts, fractions, and dexamethasone) were compared to the control group (LPS alone) by Dunnett’s test. The results were analyzed using one-way ANOVA followed by Tukey’s test. Differences with *p*-values of 0.05 or less were considered to be statistically significant. All statistical analysis was performed using GraphPad Prism v. 5.00 software (GraphPad Software, Inc., La Jolla, CA, USA).

## 4. Conclusions

In conclusion, all species evaluated showed some level of inhibition of prostanoid production. Although full chemical characterization of the studied extracts and fractions would be revealing and very useful for a better understanding of the main compounds responsible for the effects observed in the fractions and extracts evaluated in this work, the aim to assess anti-inflammatory effects of the extracts and fractions was achieved. The findings for *M. sylvestris* herein shown indicate better anti-inflammatory action if flower and leaf extracts are used together, while some COX-2 selectivity may be present. For *S. cordifolia*, more evidence of action by the cyclooxygenase pathway was provided. Although *S. cordifolia* is less used than *M. sylvestris*, the former showed better prostanoid inhibition than the latter. *P. graveolens* deserves more studies; while the findings are preliminary, it demonstrates potential to be useful based on this model of inflammation. In general, aqueous extracts or extracts rich in polar compounds are potentially more efficacious.

## Figures and Tables

**Table 1:** Yields (% dry matter, *w*/*w*) of fractions obtained from extracts of leaves.

Species	HF	CF	EAF	RF
*M. sylvestris*	48.1	13.0	11.7	27.2
*S. cordifolia*	47.7	7.8	7.0	37.5
*P. graveolens*	40.2	11.5	13.4	34.9
